# Insights into Early Interactions on Innovative Developments with European Regulators

**DOI:** 10.1007/s43441-024-00686-7

**Published:** 2024-08-30

**Authors:** David W. Uster, Valentina Cordo’, Emmanuel Cormier, Falk Ehmann

**Affiliations:** European Medicines Agency (EMA), Amsterdam, The Netherlands

**Keywords:** Innovation, Regulatory support, EMA, Drug development, Therapies, Methodologies

## Abstract

**Introduction:**

The European Medicines Agency Innovation Task Force (ITF) acts as early point of contact for medicine and technology developers to enable innovation during early drug development stages through ITF briefing meetings.

**Aim:**

To reflect on the current pace of innovation and to assess the potential of ITF stakeholder interactions, a comprehensive analysis of the ITF briefing meetings held between 2021 and 2022 was conducted with a focus on individual questions raised by the developers and the related feedback provided by the European regulators.

**Methods:**

Questions raised during ITF briefing meetings were extracted and categorised into main and sub-categories, revealing different themes across the whole medicine development process such as manufacturing technologies, pre-clinical developments, and clinically relevant questions.

**Results:**

There was positive feedback from regulators who gave initial guidance in 85% of the answers, provided concrete examples in 20% of the answers and recommended to continue discussions through additional regulatory procedures in 22% of the answers.

**Conclusion:**

This analysis frames the content and the type of topics discussed during ITF briefing meetings. Moreover, it describes the type of regulatory feedback provided to medicine developers and identified potential for improvement of these early interactions. Therefore, this analysis emphasises the role of ITF briefing meetings in fostering innovation in medicine.

## Introduction

The European Medicines Agency (EMA) is the regulatory body founded in 1995 to protect public and animal health in the European Union [1]. Within its Regulatory Science and Innovation task force, EMA has established early points of contact to facilitate communication between researchers, medicine and technologies developers, and regulators during the early stages of drug development. These regulatory support tools are designed to uphold three strategic goals of the EMA regulatory science strategy: catalysing the integration of science and technology in medicine development, enabling and leveraging research and innovation in regulatory science, and driving collaborative evidence generation and improving the scientific quality of evaluations. Early regulatory interactions play a key role in achieving these goals by engaging with medicine researchers and developers to identify scientific and regulatory gaps and possible solutions, by assisting medicine developers in the collection and analysis of high-quality data, by promoting the use of novel technologies and by contributing to EMA and European network preparedness [2].

EMA’s Innovation Task Force (ITF) [3] is a multidisciplinary group that provides a forum for early dialogue between regulators and developers of innovative medicines or technologies on scientific, regulatory and legal topics [4]. The ITF interacts with innovators through the so-called *ITF briefing meetings;* informal, 90 min brainstorming sessions that are free of charge where applicants can ask questions to European regulators and experts to receive an initial guidance on the development of their innovative products. The service is not limited to innovative medicines such as gene and cellular therapies but also includes novel methodologies and technologies such as artificial intelligence, manufacturing, drug delivery (e.g., nano-formulations) and novel approaches to replace, reduce, and refine animal use (3Rs) in medicine development as well as biomarkers.

During ITF meetings, applicants have the opportunity to discuss their developments not only with EMA representatives but also with several experts from the European medicines regulatory network. This network consists of more than 4000 experts from over fifty national competent authorities across the EU, including assessors, inspectors and members of EMA scientific committees and working parties. Relevant experts are invited by the ITF secretariat through dedicated calls for expression of interest based on type of expertise needed for each meeting. Moreover, regular communication with EMA committees and working parties ensures that their members are aware of upcoming ITF briefing meetings. Interested experts can request to attend one or more meetings by providing their CV and their updated declaration of interests. Furthermore, additional experts can be recruited. Experts can provide not only regulatory guidance but also their scientific expertise [5].

Through these early interactions EMA aims to facilitate the transition from early stages of medicine development to marketing authorisation application by reflecting on the innovation and preparing the applicant for subsequent procedures like scientific advice [6], qualification of novel methodologies [7], and marketing authorisation application [8].

Furthermore, such early dialogues can uncover the need for specialised expertise on the regulatory side and address the impact of emerging innovations on the European regulatory system, and thus prepare the European medicines regulatory network for the future. For this purpose, interactions between EMA and medicine developers are periodically analysed to identify emerging technologies or trends in medicine research and development [9–11].

The aim of this paper was to present a systematic analysis of all the questions posed by medicine and technology developers during the ITF briefing meetings held between 2021 and 2022, and identify areas in need of additional regulatory support. Moreover, we analysed the type of answers and feedback provided by regulators in order to evaluate the possible impact of these informal early interactions on the medicine development process.

## Methods

The data source for the analysis described in this paper were the ITF briefing meetings conducted between 1 January, 2021 and 31 December, 2022. The documentation related to such interactions (ITF request form and meeting minutes) were systematically collected and reviewed. All meetings accompanied by minutes were included in the analysis, regardless of the type of applicant or type of innovative development discussed. Interactions without recorded minutes (e.g., short clarification calls) were excluded from the analysis.

For statistical purposes, information on the applicants and the innovative development discussed (type and stage of innovative development as well as the enabling technology labels [10]) were extracted from the ITF request forms and graphically summarized.

Similarly, all meeting minutes were screened for the questions posed by the applicants. Each question was first assigned to a *main category* that represented the phases of healthcare/medicine development (i.e. ‘Chemical, manufacturing and controls’ (CMC), pre-clinical, clinical development, and others). Subsequently, the questions in each main category were assigned to at least one of the two subcategories such as *scientific aspects* (e.g., biomarker, mechanism of action, etc.) and *regulatory aspects* (e.g., assistance with product classification, regulatory procedures, etc.), respectively (Table 1).

**Table 1:** Overview of the Main Categories of the Questions and Their Corresponding Subcategories. Each Category is Provided with a Short Description and/or Examples of Topics Included.

Main Category	Subcategory	Description	Exemplary Questions
	Scientific aspects		
Chemistry, manufacturing, and controls (CMC)	Analytical technology	Novel analytical technologies, data collection, microbiological monitoring	What kind of interactions along the project could be set up with regulators regarding implementation strategy as part of an advanced process control strategy? What are the experts’ suggestions on the outlined strategy to show equivalence or superiority with the proposed new methodology?
	Audit	Audit process	How are site audits conducted during pandemic?
	Drug delivery systems	Combination packaging, packaging / storage conditions	–
	Good manufacturing practice (GMP)	Cross-contamination, contamination and mix-up risk analysis, innovative solutions	What kind of interactions along the project could be set up with regulators regarding specific GMP aspects?
	Manufacturing technology	Novel manufacturing technologies/design (e.g., 3D (bio-)printing, genetically modified organisms, multi-facility pooling for advanced therapy medicinal products)	What are the experts’ suggestions on the applicable guidelines for 3D bioprinting?
	Materials	(bio-) material related questions (e.g., for organoids, printing, novel raw materials)	Can the proposed new product be classified as tissue engineered product?
	Quality	Impurities, quality management system, quality control	What are the experts’ opinions/suggestions on the proposed approach how to monitor the quality of a medicinal product?
	Stability	Stability of the product	–
	Validation	Validation strategy for novel technology	What are the expert’s opinions on the acceptability of the proposed contamination control strategy?
Pre-clinical	Analytical technology	Novel analytical technologies, data collection	What kind of interactions along the project could be set up with regulators regarding this analytical technology?
	Biomarker	Pharmacogenomics, novel biomarker development	Do experts have any suggestions on the applicants’ strategy to identify novel biomarker in the particular disease area?
	Efficacy	Level of evidence needed	Can experts provide guidance on the acceptability of the discussed assay?
	Materials	Use of novel materials in pre-clinical stages	What are the experts’ suggestions for the use of novel materials in pharmaceutical preparations?
	Mechanism of action	Questions related to biochemical interaction and pharmacological effect (e.g., kill switches, mode of action of new molecule, ligand…)	In the experts’ opinion, would the proposed strategy provide sufficient demonstration of a functional kill switch?
	Proof of concept	.. of novel medicinal products (e.g., advanced therapy medicinal products) animal models (adequacy/reduction), pharmacology studies	Can experts comment on the proof of concept-strategy for the proposed advanced therapy medicinal product?
	Safety/toxicity	Toxicity studies, product safety, immunogenicity	Can regulators comment on regulatory landscape for existing immunosuppression protocols?
	Study design	Design of the pre-clinical development (3Rs, animal studies (adequacy/reduction), design of database)	What are the experts’ opinions and recommendations for the design of a database for collecting preclinical data?
Clinical	Biomarker	Pharmacogenomics, novel biomarker development	Can experts comment on possible future interactions to discuss new clinical outcome measures?
	Dosage forms	Flexible dosage range (in terms of personalized medicine)	What is the experts’ view on the proposed flexible dosage forms?
	Efficacy	Potency assay, level of evidence needed	What is the experts’ opinion on the level of evidence needed?
	Endpoints	Novel clinical endpoints (also using digital health technologies)	How to best interact with regulators to discuss the use of digital health technologies-derived endpoint for regulatory decision making?
	Safety/toxicity	Off target risk and shedding (gene therapy related)	What suggestions do the experts have for monitoring patient safety once gene therapy/treatment is initiated?
	Study design	Design of the clinical development (first in human, technology package, method of enrolment, metadata sharing, real world data, platform trials)	What regulatory advice can be obtained with regard to regulatory pathway and requirements under the latest in-vitro diagnostic regulation?
	Validation	Validation strategy of technology/biomarker for use in clinical study, requirements	What are the experts’ opinions on the proposed clinical validation plans?
Other		Novel ways of drug repurposing, novel data exchange platform, topics not covered by any other category	Can experts comment on the proposed data platform?

	Regulatory aspects		
Chemistry, manufacturing, and controls (CMC)/Pre-clinical/Clinical/Other	Digital tools	Tools supporting the innovation/product/methodology/technology; complements enabling technology category (E/M health) as declared by applicant; e.g., digital health technology (like personalized medicines applications), digitalization in manufacturing, endpoint derivation	How could sponsors drive innovation within clinical trials with regard to data integrity using blockchain methods?
	Product classification	Medicinal product vs medical device; companion diagnostics; Tissue engineered product; advanced therapy medicinal products (ATMP); orphan drug, etc.; Hospital Exemptions (HE) under remit of NCA	Does classification of the proposed product fall under the remit of EMA or national competent authorities?
	Product labelling	Novel summary of product characteristics (e.g., digital/applications), more nuanced product labelling (through pharmacogenomics)	–
	Qualification of novel methodologies	The qualification process addresses innovative drug development methods and tools focusing on the use of novel methodologies developed by consortia, networks, public/private partnerships, learned societies and pharmaceutical industry for a specific intended use in research and development of pharmaceuticals	Can experts comment on the next steps to initiate the qualification process?
	Recommendation on follow-up	Drafting of white paper/position paper, involvement of notified bodies, regulatory interaction plan	Can experts comment on potential collaboration between applicant and regulatory agency?
	Regulation gap	Applicant is missing guideline, regulatory category unclear	Can experts refer to guidelines for products like organs-on-chip?
	Regulatory procedures	Lack of knowledge about existing documents (e.g., how to apply for PRIME, ATMP classification…), Regulatory pathway (e.g., paediatric investigation plan) for innovation unclear to applicant	What are recommended next steps to request PRIME eligibility for the presented product?

– Examples questions could not be provided due to the confidential nature of the proposed topics.

Finally, the responses of the experts were extracted and categorised according to the type of feedback provided (Table 2). The assigned labels were cross-checked for consistency and accuracy.

**Table 2:** Labels of the Answers.

	Type of Answers	Percentage [%]	Description
+	Supportive feedback	16	Experts generally appreciate approach/perspective/suggestion
	Specific documents/guidelines/regulations recommended	22	Experts pointed out to specific documents/guidelines/etc
	Suggestions/recommendation made	37	Experts made concise suggestions
	Examples given	21	Named concrete examples
	Post-meeting notes added	16	Identified by searching for terms: “postmeeting” or “post-meeting”
~	Diverging feedback	3	Experts display different views on the topic
	Questions raised	8	ITF raised multiple questions which could be answered ad hoc by applicant
	Clarification/More details requested	12	ITF requests more data/information to appropriately address the question
	Referred to other procedure	22	Experts refer applicant to other functions like scientific advice, national competent authorities (either directly or after giving initial advice)
o	Deviating feedback	3	Experts recommend an alternative/do not (fully) agree with approach/perspective/suggestion
	Postponed	3	Question was not addressed during the meeting

Percentage contains the occurrence of the labels relative to the total number of answers (N = 275). More than one label can be assigned to an answer. Thus, sum of proportions might be higher than 100. Description contains a short explanation of the label and/or examples.

*ITF* innovation task force.

The data preparation and all statistical and graphical evaluation were done using Microsoft Excel and R (Version 4.1) [12].

## Results

**Figure 1:**
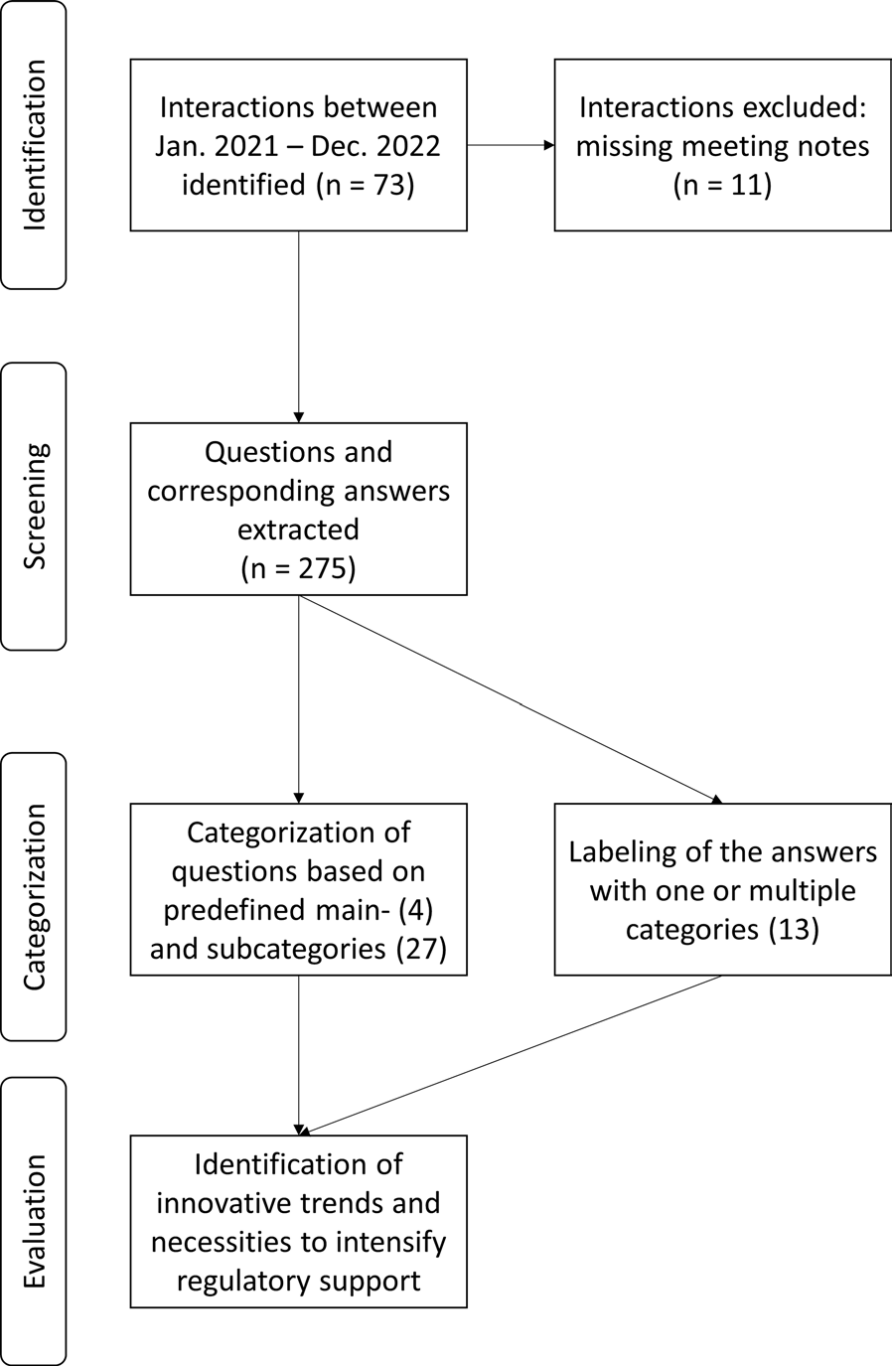
Workflow of the Analysis of the ITF Briefing Meetings Held between 2021 and 2022 at the European Medicines Agency. The Analysis Consisted of Four Steps: Identification of Interactions, Screening of the Meeting Minutes, Categorisation of the Question and Answers and Evaluation of the Content.

**Figure 2:**
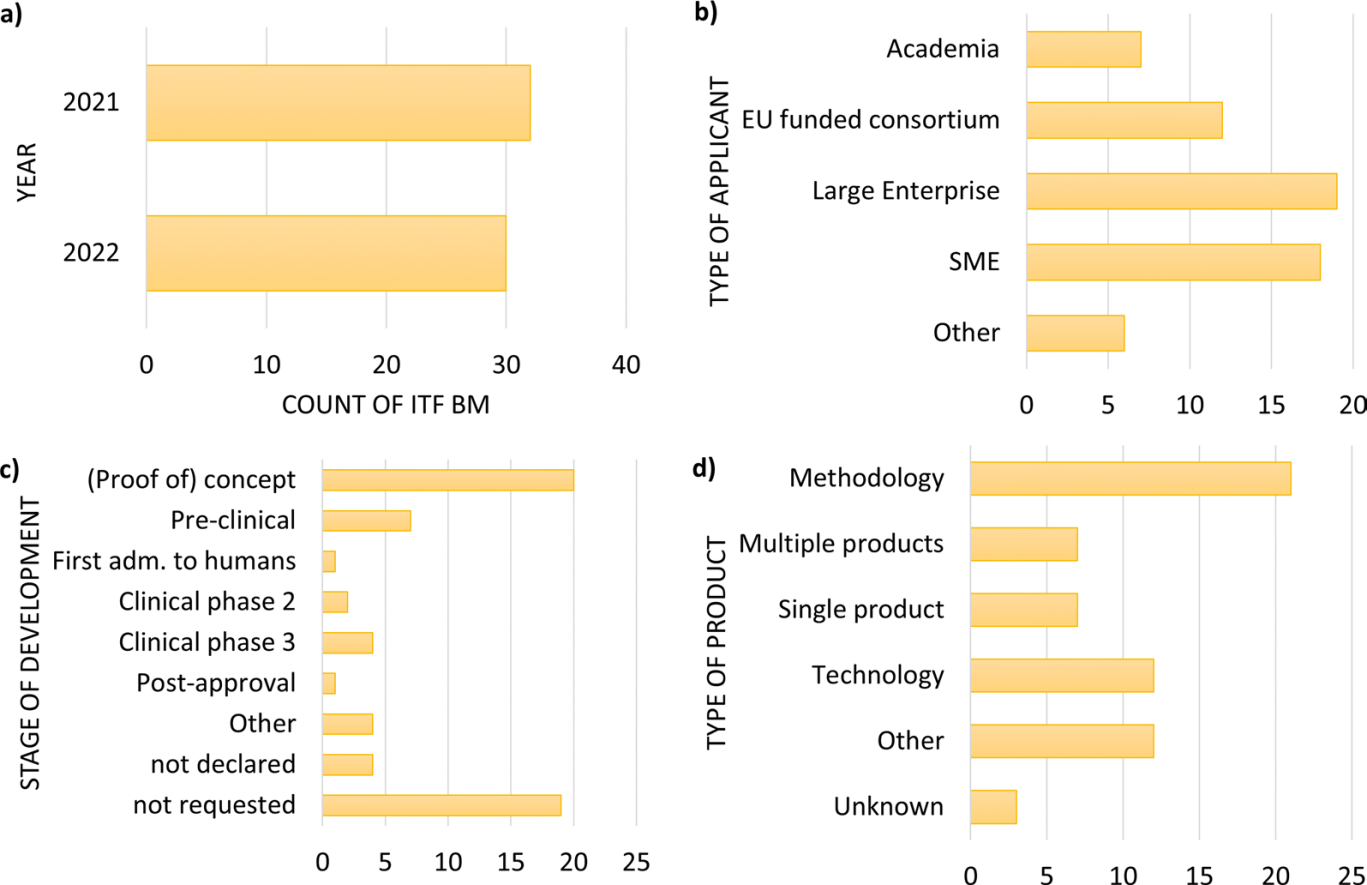
Innovation Task Force Briefing Meetings (ITF BM). **a** Number of ITF BM per Year. **b** Number of Applicants According to Their Type. SME—Small and Medium Enterprise **c** Number of Applicants Based on the Stage of Development Declared in the Application Form. Single Product—One Novel Medicinal Product (e.g., New Chemical Entity, Biologic, Advanced Therapy Medicinal Product); Multiple Products—for Example, Medicinal Products that Share Common Elements (e.g., Platform Approaches, Class of Molecules); Methodology—for Example, Novel Approach Methodologies (e.g., Related to 3Rs Principles Such as Organ-on-Chip, In Vitro Assays), Statistical Method or Analysis, Clinical Trial Methodology; Technology—for Example, Digital Tool, Software or Application of Blockchain for Data Integrity. The Stage of Development was “*Not Requested*” in 19 ITF Briefing Meetings Before an Updated Application Form was Implemented in Early 2021. adm—Administration. **d** Number of Applicants Stratified by Type of Product Discussed.

**Figure 3:**
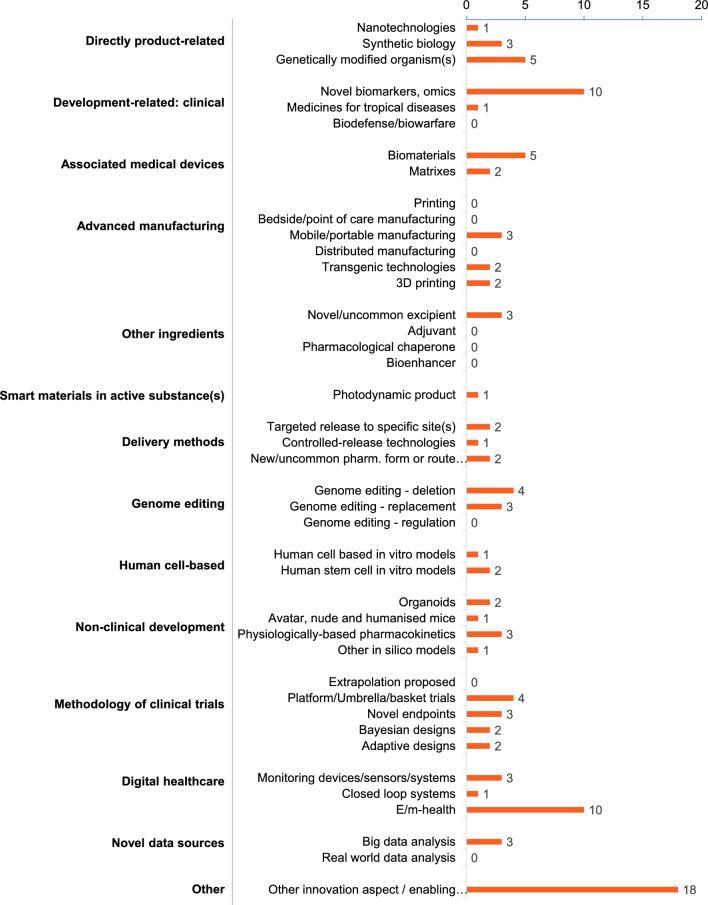
Enabling Technology Labels of the Innovations Discussed in the 62 ITF Briefing Meetings. Up to Three Labels per Innovation were Chosen by the Applicant. The Complete List of Labels is Provided by the Innovation Task Force in the Application Form [12].

**Figure 4:**
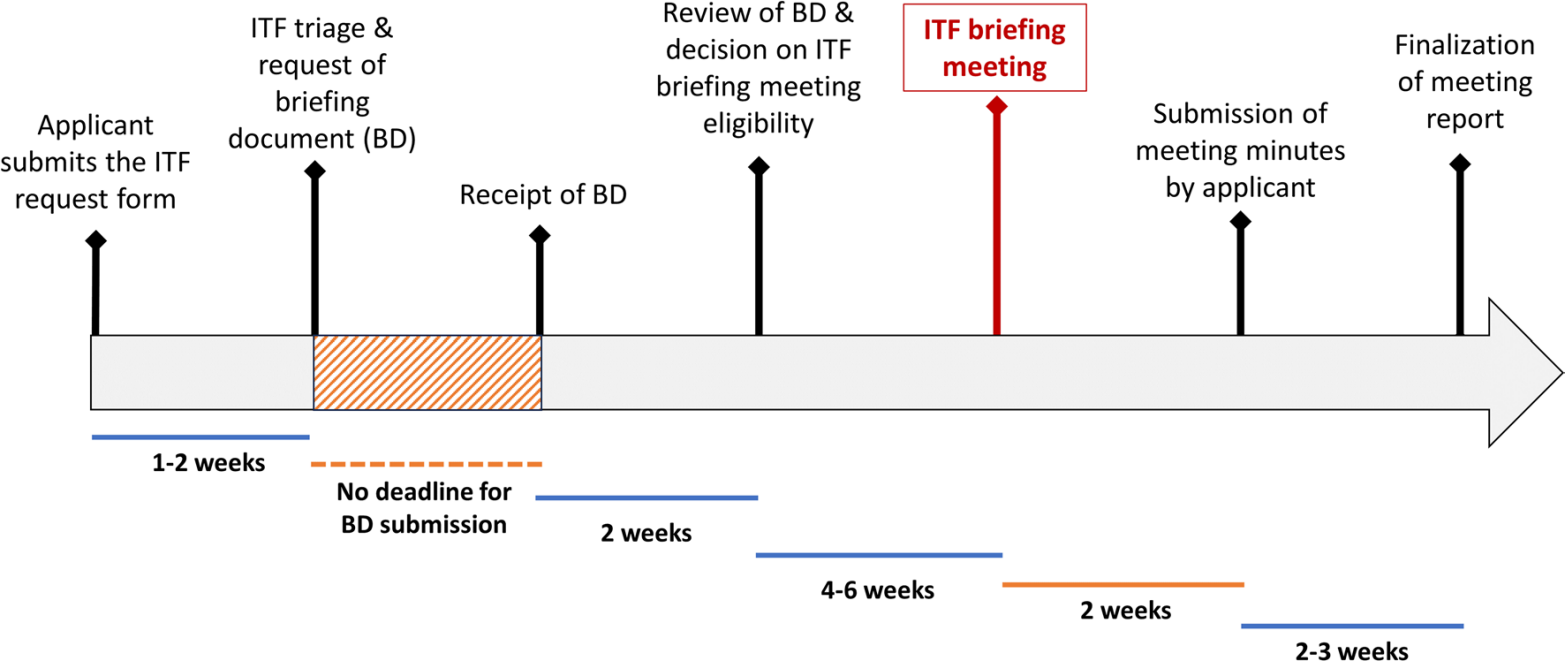
Process Chart Displaying the Timeline from Submission of the Application for an Innovation Task Force Meeting to the Conclusion of the Interaction. Blue Bars Indicate Timings Mainly Influenced by the Regulator While Orange Bars Indicate Steps Depending on the Applicant.

The systematic collection of ITF briefing meetings held between 1 January, 2021 and 31 December 2022 resulted in a total of 73 interactions, of which 11 were excluded from the subsequent analysis since meeting minutes were not available (Fig. 1), mostly due to the more informal nature of some meetings (e.g., brief call with EMA representatives only). From the remaining 62 ITF briefing meetings, 32 meetings were held in 2021 and 30 in 2022 (Fig. 2a). Large enterprises took part in the meetings as often as small and medium enterprises (SME) with n = 18 and n = 19, respectively (Fig. 2b). Moreover, academic applicants and representatives of European funded consortia also discussed their innovations in ITF briefing meetings, although less frequently (n = 7 and n = 12, respectively; Fig. 2b). Of all applicants, a quarter declared to be at proof-of-concept stage with their development (Fig. 2c). The type of development most frequently discussed during these meetings was *methodology*, followed by *technology* (Fig. 2d) [13]. In general, the topics were very diverse and encompassed a broad range of subjects across the whole drug development process, from the pre-clinical stage (genome editing, genetically modified organisms, biomaterials) to clinical development (biomarkers and omics, clinical trial design and digital healthcare) as illustrated in Fig. 3. Applicants can apply at any time for an ITF briefing meeting. Figure 4 describes the timeline from the submission of application to conclusion of the interaction [14].

To get a better insight in the type of questions asked by applicants and to evaluate the type of feedback provided by regulators, all the questions (n = 275) and their related answers were extracted from the 62 ITF briefing meeting reports. Examples of generic questions addressed during ITF meetings are listed in Table 1. Each meeting discussed between one and seven topics, with a mean of 4.4 questions (range 2–8) being addressed per meeting.

**Figure 5:**
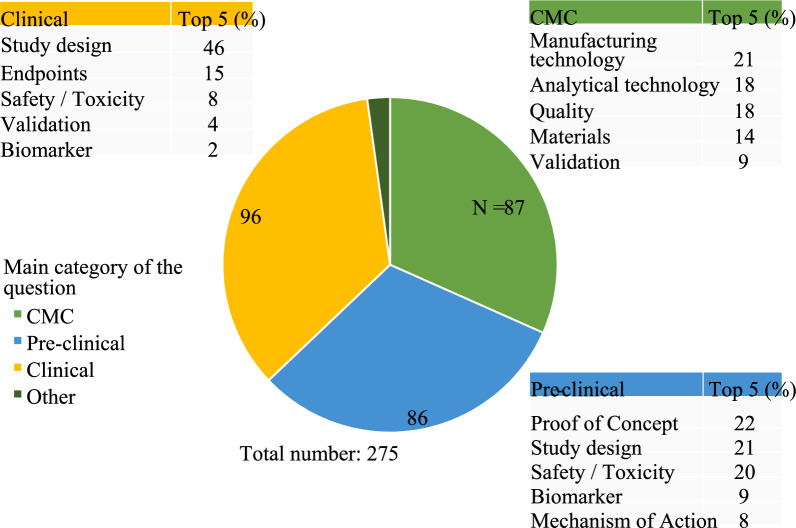
Categorisation of the Questions (N = 275) Raised During the Interactions into the Four Main Categories. Numbers in the Pie Chart Represent the Number of Questions Assigned to the Respective Main Category. The Coloured Tables Represent the Five Most Frequently Chosen Scientific Subcategories per Main Category Displayed as Percentage of the Main Category, Respectively.

For the questions, three main categories (CMC, pre-clinical, clinical) and a total of 24 scientific and 7 regulatory/procedural subcategories were identified (Fig. 5). Answers provided by the experts were classified with one or multiple labels based on the type of feedback provided as displayed in Table 2. In total, 11 labels were assigned to the answers, with an average of 1.61 (range 1–4) labels per answer.

Slightly fewer questions related to CMC (n = 87) and pre-clinical (n = 86) categories were seen compared with questions in the clinical category (n = 96), while six questions could not be classified into any of the three main categories. Examples of such questions were topics related to drug repurposing and novel data exchange platforms. Thus, these questions were included in the *other* category (Fig. 5).

The most recurring subcategories (i.e. the top 5 in percent) for each of the four main categories are displayed in Fig. 5. Manufacturing and analytical technologies were the most common scientific topics within the CMC main category, proof of concept and study design for the pre-clinical domain, and study design and endpoints for the clinical main category, respectively. For 20% of the questions, regulators provided concrete examples, including references to specific publicly available scientific and regulatory documents (e.g., guidelines, reflection papers or literature). In 22% of the feedbacks provided, experts recommended to continue their engagement with the regulators through either another procedure at EMA, such as scientific advice or qualification of novel methodologies (n = 38 and 4, respectively), or through national competent authorities’ regulatory advice (n = 10) [6]. Even though experts highlighted that to receive additional feedback and advice on the topic proposed, a different regulatory procedure should be undertaken, actionable initial guidance was provided in 85% of the cases. Moreover, for 16% of the questions, a clarification in writing was requested by the applicant through the ITF briefing meeting report which was followed by a written post-meeting explanation. In rare cases (3% of the answers), the different experts present at the meeting expressed diverse views and opinions on the same topic instead of a consensus (classified as *diverging feedback*) or did not agree with the applicant’s proposed plan or strategy (i.e. *deviating feedback*). Of note and relevance, ITF experts were reciprocally engaged by asking questions to further explore additional aspects of the proposed topics and to better reflect on potential path forward for the development of the product or technology presented.

## Discussion

Facilitating and enabling innovation in medicines is a key strategic goal and ambition of the EMA and European Medicines Agency Network strategies [15]. This analysis provides insight into early interactions with European regulators by analysing 62 ITF briefing meetings, with a focus on the questions discussed and feedback provided.

First, questions encompass all the stages of medicine development from proof-of-concept to clinical stage. For example, early developmental topics ranged from novel air monitoring methods in manufacturing facilities to biotechnical kill switches to ensure safety of novel cell and gene therapies [16, 17]. Topics related to later stages of medicine development included discussions on study design strategies and novel clinical endpoints being digitally derived using artificial intelligence.

In general, there was positive and supportive feedback from regulators that stimulated an open and constructive dialog. Often concrete examples and suggestions on which guidelines to follow for each case were provided (Table 2) to support the applicant. Such type of feedback can be very helpful since developers often find it challenging to identify the guidelines most relevant to their development. This is particularly the case for innovative products for which the current regulatory framework may not be well suited. The challenge may be even more pronounced for SMEs and academic applicants who are often not familiar with regulatory requirements along the medicine lifecycle.

Additionally, having informal discussions has multiple advantages. General brainstorming enables both applicants and regulators to freely weigh several options against each other without the absolute necessity to agree on a single consensus at such early stage. Nonetheless, applicants can expect valuable input given the broad range of expertise present within the European regulatory network. Regulators can access the meeting documentation in advance of the meeting, analyse it and prepare answers. Moreover, constructive feedback and spontaneous questions from regulators can help applicants reflect on their strategy and have a positive impact on their product development. It is expected that early interaction with regulators could lead to faster development and increased chance of successful applications [18, 19]. When medicines developers become familiar with the regulatory system and requirements, there can be a higher compliance to the recommended standards. Such compliance can be achieved through planned engagements with regulators (e.g., scientific advice and pre-discussion meetings) at important steps along the whole medicine development process [9, 19–21].

The challenges faced when categorising the questions and answers need to be acknowledged. In fact, agreement on label definitions (Tables 1 and 2) may entail subjective categorisation. To minimize the impact, multiple team meetings were conducted to agree on special cases as well as on appropriate categorisations. Furthermore, the number of questions per meeting (range 2–8) was usually greater than the number of topics (i.e., subcategories) discussed per meeting (range 1–7). This could indicate a certain clustering or enrichment of subcategories based on the focus of the meeting that depends on the stage of development and challenges encountered by the applicant. Given that ITF meetings are intended to offer a flexible type of interaction to support the needs of medicine and technology developers, the risk of a certain cluster bias cannot be excluded.

In general, categorisation requires consistency of use throughout the various interactions with regulators to ensure a meaningful assessment of impact. Therefore, to analyse the journey of innovation a consistent approach throughout the lifecycle of a product development is necessary. Furthermore, to fully evaluate the beneficial value of interactions between regulators and medicines developers, an assessment that includes the stakeholders’ perspectives should be put in place (e.g., post-meeting satisfaction survey for participants and monitoring of follow-up regulatory interactions).

The representative interactions above indicate the opportunities offered by early and low-threshold interactions for both applicants and experts. While the exchange of information at early developmental stages can certainly impact the applicant’s strategy, it also provides valuable and early insights to regulators. Capability and capacity needs on specific topics can be easily identified as well as the necessity to update or develop new supportive guidelines. Furthermore, providing general guidance towards more formal follow-up procedures (like scientific advice) will favour a streamlined approval process of medicinal products [18, 19].

## Conclusion

The current analysis describes for the first time the type of questions and answers from the ITF briefing meetings held in 2021 and 2022. Although a formal measurement of the long-term impact is still to be implemented (e.g. by initiating a follow-up strategy or systematic survey to monitor the regulatory actions following the ITF briefing meeting), the current reflections demonstrate the variety of topics presented by medicine developers, and indicate the type of feedback that can be offered by regulators. A continuation and enhancement of this dialog is recommended to ultimately strengthen the European innovation ecosystem.

## Disclaimer

The views expressed in this article are the personal views of the author(s) and may not be understood or quoted as being made on behalf of or reflecting the position of the regulatory agency/agencies or organisations with which the author(s) is/are employed/affiliated.

## Data Availability

No datasets were generated or analysed during the current study.
